# Typical histological pattern of seminoma and its immunoreactivity: A diagnostic challenge

**DOI:** 10.1002/ccr3.4091

**Published:** 2021-05-24

**Authors:** Nafisa Abedin, Bishnu Pada Dey

**Affiliations:** ^1^ Department of Pathology Bangabandhu Sheikh Mujib Medical University (BSMMU) Dhaka Bangladesh

**Keywords:** histological pattern, seminoma

## Abstract

A 20‐year‐old man presented in the Department of Urology, with the complaint of left‐sided lower abdominal lump for past 9 months. The patient denied of having any constitutional symptoms including weight loss.

## Graphical abstract

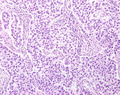

Although seminoma is a common tumor of testis, occasionally it can be a diagnostic challenge! Typical histological pattern and immunoreactivity of the tumor cells play a pivotal role in the diagnosis of this tumor. The presented images provide the typical histomorphological features of seminoma and immunopositivity of the tumor cells.

Physical examinations revealed a firm‐to‐hard, nontender, well‐defined mass in the left lower abdomino‐pelvic region with empty left scrotum. However, right scrotum was in situ position. MRI of pelvis showed an irregular, soft tissue mass measuring about 5 cm × 3 cm × 3 cm in the left pelvic region. This patient was diagnosed as a case of intra‐abdominal tumor in undescended left testis. The patient was operated with excision of the pelvic mass. Subsequently, the excised mass was examined in the Department of Pathology along with histological analysis.

A conclusive diagnosis of seminoma requires meticulous histopathological and immunohistochemical analyses. Better understanding of the microscopy findings and the immunoreactivity of these tumor cells makes the diagnosis easier Figure 1.

**FIGURE 1:**
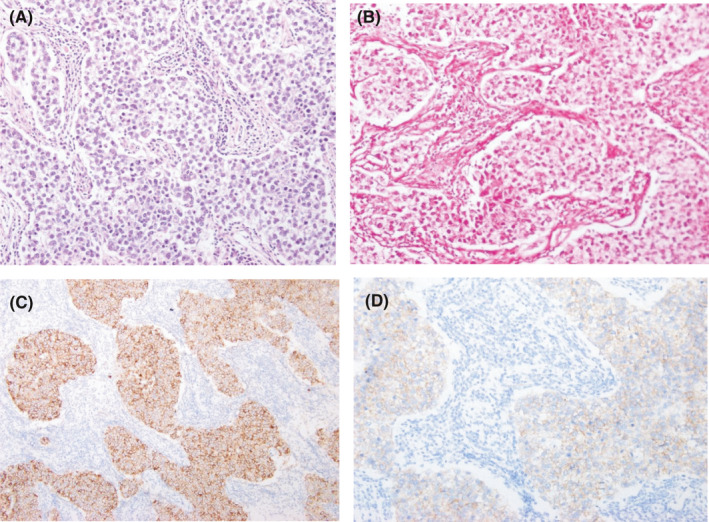
Histology of seminoma. A, Classical seminoma, with uniformly distributed tumor cells arranged in lobules, demarcated by dense fibrous septae infiltrated by lymphocytes, H&E ×100. B, Large polygonal neoplastic cells, with clear, glycogen‐rich cytoplasm, PAS ×100. C, Tumor cells are reactive to immune stain placental alkaline phosphatase ×100. D, Tumor cells are reactive to immune stain c‐Kit ×100^1^

## CONFLICT OF INTEREST

There is no conflict of interest among the authors.

## AUTHOR CONTRIBUTIONS

NA: collected data and reviewed literature and wrote the manuscript. BPD: revised the manuscript and supervised.

## ETHICAL APPROVAL

Hereby, I, Dr Nafisa Abedin consciously assure that for the manuscript, the following is fulfilled: 1) This material is the authors' own original work, which has not been previously published elsewhere. 2) The paper is not currently being considered for publication elsewhere. 3) The paper reflects the authors' own research and analysis in a truthful and complete manner. 4) The paper properly credits the meaningful contributions of co‐authors and coresearchers. 5) The results are appropriately placed in the context of prior and existing research. 6) All sources used are properly disclosed (correct citation). Literally copying of text must be indicated as such by using quotation marks and giving proper reference. 7) All authors have been personally and actively involved in substantial work leading to the paper and will take public responsibility for its content. The violation of the Ethical Statement rules may result in severe consequences. I agree with the above statements and declare that this submission follows the policies of Solid State Ionics as outlined in the Guide for Authors and in the Ethical Statement.

## Data Availability

The data that supports the findings of this study are openly available in Google Scholar with the identifier http://doi.org/10.1053/j.semdp.2005.11.003.
